# Paternal De Novo Variant of *TAOK1* in a Fetus With Structural Brain Abnormalities

**DOI:** 10.3389/fgene.2022.836853

**Published:** 2022-07-19

**Authors:** Lihua Yu, Chaoxiang Yang, Ning Shang, Hongke Ding, Juan Zhu, Yuanyuan Zhu, Haowen Tan, Yan Zhang

**Affiliations:** ^1^ Medical Genetics Centre, Guangdong Women and Children Hospital, Guangzhou, China; ^2^ Department of Radiology, Guangdong Women and Children Hospital, Guangzhou, China; ^3^ Department of Ultrasound, Guangdong Women and Children Hospital, Guangzhou, China; ^4^ Aegicare (Shenzhen) Technology Co., Ltd., Shenzhen, China

**Keywords:** dilated lateral ventricle, *TAOK1*, trio-whole exome sequencing, rare disease, neurodevelopment disorder

## Abstract

A dilated lateral ventricle is a relatively common finding on prenatal ultrasound, and the causes are complex. We aimed to explore the etiology of a fetus with a dilated lateral ventricle. Trio whole-exome sequencing was performed to detect causative variants. A *de novo* variant of *TAOK1* (NM_020791.2: c.227A>G) was detected in the proband and evaluated for potential functional impacts using a variety of prediction tools. Droplet digital polymerase chain reaction was used to exclude the parental mosaicism and to verify the phasing of the *de novo* variant. Based on peripheral blood analysis, the parents did not exhibit mosaicism at this site, and the *de novo* variant was paternally derived. Here, we describe a fetus with a *de novo* likely pathogenic variant of *TAOK1* who had a dilated lateral ventricle and a series of particular phenotypes. This case expands the clinical spectrum of *TAOK1*-associated disorders. We propose a method for solving genetic disorders in which the responsible genes have not yet gone through ClinGen curation, particularly for prenatal cases.

## Introduction

The TAO kinase family consists of three genes, *TAOK1*, *TAOK2*, and *TAOK3*, which encode TAOK1, TAOK2, and TAOK3, respectively (Dan et al., 2001; Miller et al., 2019). TAO kinases play multifunctional roles in many molecular and cellular events and can regulate neuronal survival and development in the nervous system (Fang et al., 2020; Hu et al., 2021). *TAOK1* is highly expressed in the human brain and plays a role in the establishment of neuronal polarity, neuronal differentiation, and early brain development (Biernat et al., 2002; Timm et al., 2006; Draviam et al., 2007; Breuss and Keays, 2014; Poon et al., 2016). Many studies have provided evidence that *TAOK1* dysfunction can result in neurodevelopmental disorders (NDDs) (Cooper et al., 2011; Xie et al., 2016; Deciphering Developmental Disorders Study, 2017; Dulovic-Mahlow et al., 2019; Satterstrom et al., 2020; van Woerden et al., 2021; Hunter et al., 2022). However, dysfunction of this kinase in prenatal cases has not been reported.

Here, we report structural brain abnormalities in a fetus with a *de novo* variant of *TAOK1*. To our knowledge, this is the first report of *TAOK1* dysfunction as a prenatal diagnosis.

### Case Presentation

**FIGURE 1:**
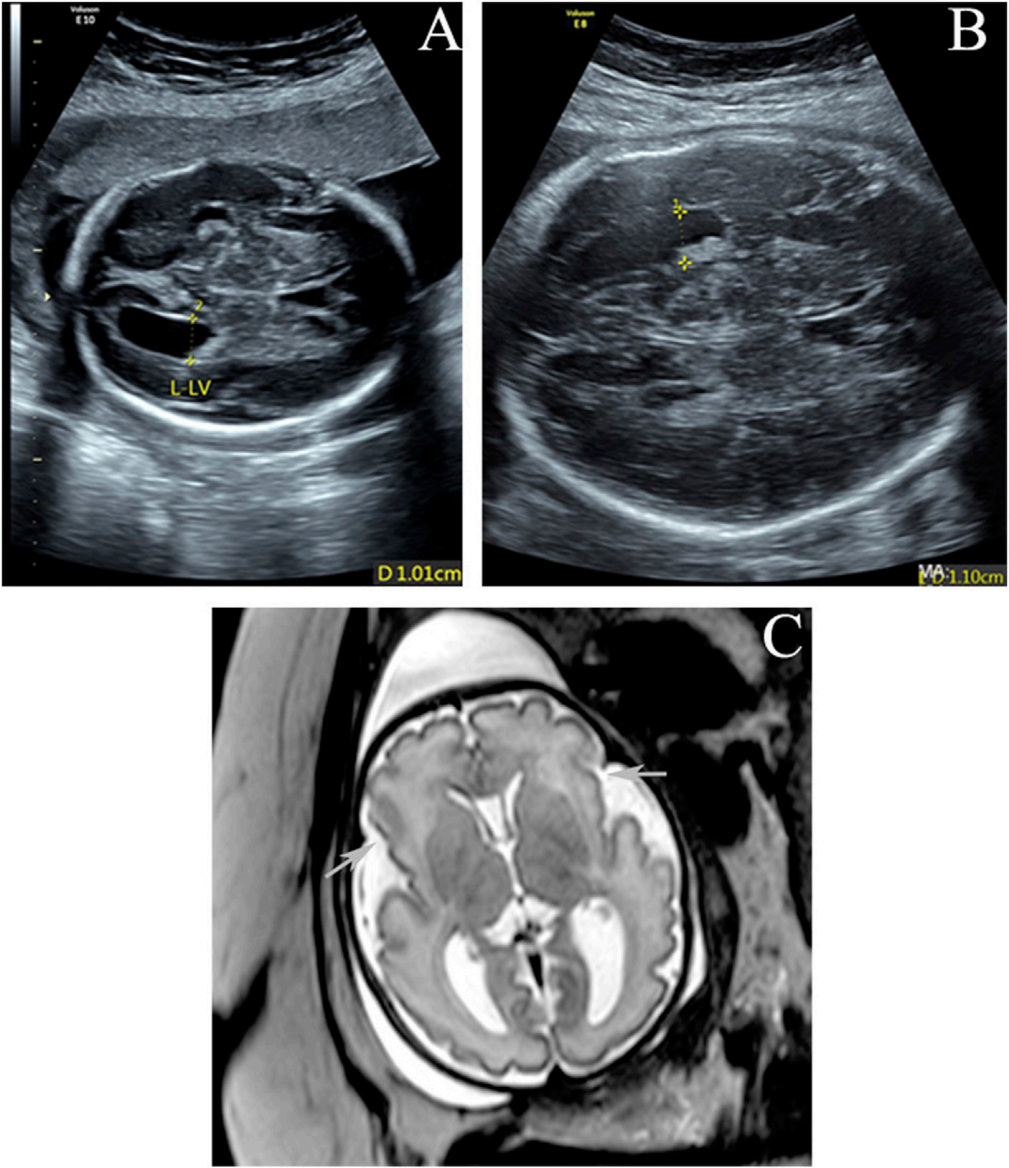
**(A,B)** Enlarged left lateral ventricle (10.1 and 11 mm) at 25 and 31 gestational weeks. **(C)** Axial T2-weighted imaging at 32 weeks of gestation shows poor bilateral and frontal operculum formation and shallow bilateral lateral fissures (arrows). The left ventricle is slightly wider.

A healthy 32-year-old gravida 3, para 2 (G3P2) woman underwent a prenatal examination at Guangdong Women and Children Hospital. She delivered two normal male infants, in 2013 and 2018, through uncomplicated vaginal deliveries. At 25 weeks of gestation for the current pregnancy, routine ultrasound scanning showed that the left lateral ventricle of the fetus was widened (10.1 mm compared to a reference of <10 mm) (Figure 1A). Common factors, such as infection and anemia, were ruled out, and COVID-19 nucleic acid tests were negative. Noninvasive prenatal testing (NIPT) indicated a low risk of fetal trisomy 13, 18, and 21. At 31 weeks of gestation, ultrasound scanning showed slight widening of the left lateral ventricle (11.0 mm compared to reference of <10 mm), with the umbilical cord surrounding the neck, of the fetus (Figure 1B). As shown in Figure 1C, magnetic resonance imaging (MRI) revealed poor bilateral and frontal operculum formation and shallow bilateral lateral fissures, which were more obvious on the right side. Bilateral polymicrogyria of the lateral fissure area could not be ruled out. The left ventricle was slightly wider. No abnormalities were observed in the corpus callosum, septum pellucidum, cerebellar vermis, or posterior fossa. Due to the abnormal cortical structure detected by MRI, interventional prenatal diagnosis was performed, along with chromosomal microarray analysis (CMA) and trio whole-exome sequencing (trio WES), simultaneously. The CMA result was negative, but trio WES detected a *de novo* missense variant of *TAOK1* in the fetus. Whole-genome sequencing (WGS) and droplet digital polymerase chain reaction (ddPCR) was then performed to identify the source of the variation. Finally, the *de novo* variant of *TAOK1* was found to originate from the paternal allele.

## Methods and Results

### Trio Whole-Exome Sequencing

Genomic DNA was extracted from amniotic fluid and peripheral blood from the fetus and parents, respectively, using the QIAamp DNA Mini Kit (Qiagen), following the manufacturer’s instructions. Trio WES was performed to detect genetic variants (see Supplementary Methods), and a *de novo* variant of *TAOK1* [GRCh37/hg19 chr17: 27802710, NM_020791.2: c.227A>G (p.Glu76Gly)] was found. No other variants were considered to contribute to the phenotype.

**FIGURE 2:**
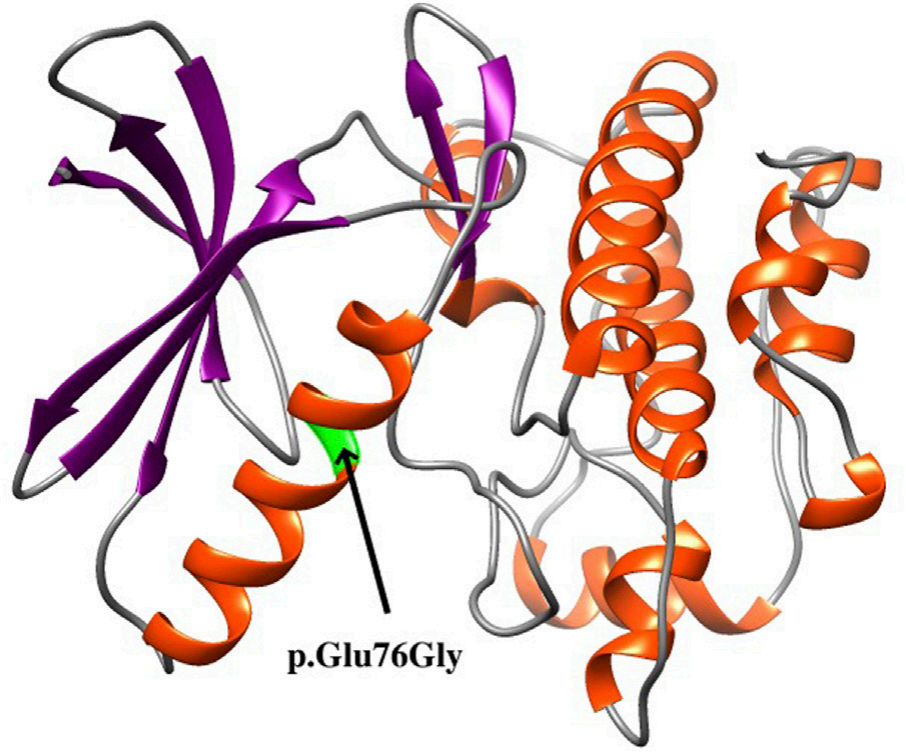
Structure of the kinase domain of the TAOK1 protein from amino acids 28 to 281, showing the localization of the identified variant p.Glu76Gly.

The *de novo* variant c.227A>G is absent in the general population according to public databases (gnomAD, 1000 Genomes Project, NHLBI Exome Sequencing Project 6500, and Exome Aggregation Consortium). This variation has not been previously reported in the ClinVar or PubMed databases (retrieved 15 January 2022). A variety of prediction tools (SIFT, DANN, and REVEL) were used to evaluate the possible functional impact of c.227A>G, and it is predicted to be a damaging variation by all three tools. Furthermore, various algorithms (GERP, phyloP, phastCons, and SiPhy) and multiple sequence alignments from the UCSC genome browser predicted that this position is conserved across multiple vertebrate species (from zebrafish to human). The variant p.Glu76Gly is located in the “Protein kinase” domain of TAOK1 (UniProt ID #Q7L7X3) in which benign variants are not found in ClinVar database. Although the protein structure of TAOK1 was not available in the PDB database, the structure of the kinase domain (amino acids 28–281) was predicted with the I-TASSER server, as illustrated in Figure 2 (Roy et al., 2010). The model with the highest confidence (C-score) and topological similarity (Tm-score) is used. The identified variant p.Glu76Gly is predicted to be located in an alpha-helix of the protein (Figure 2).

### Whole-Genome Sequencing

To identify the phase of the *de novo* variant, WGS was performed for the proband (see Supplementary Methods). A heterozygous variant, c.306+468G>T (GRCh37/hg19 chr17: 27803257G>T), in *TAOK1* was found, which is 547 bp downstream of c.227A>G and was used as the reference for ddPCR analysis.

**FIGURE 3:**
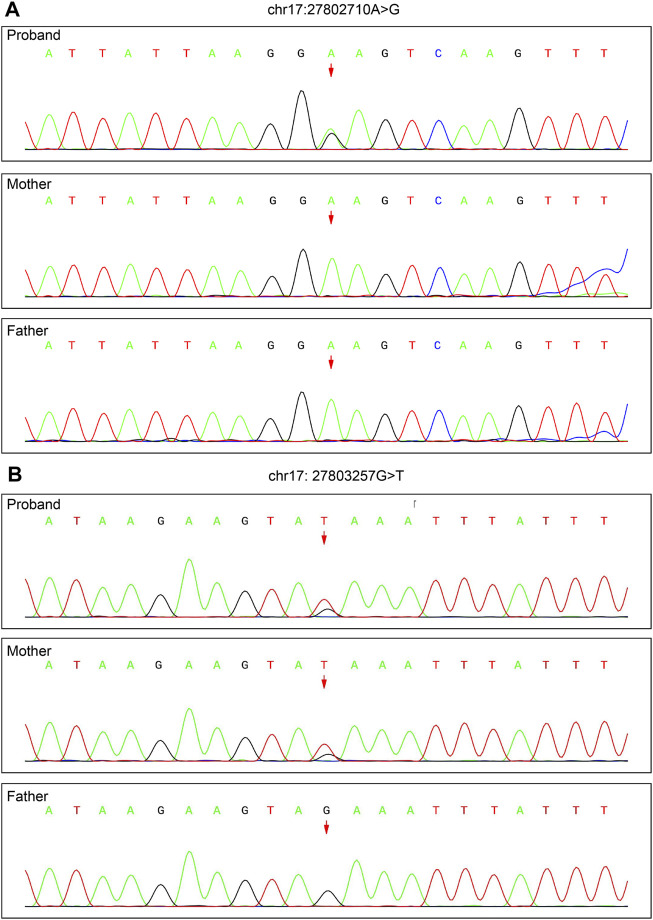
Validation of the *de novo* variant c.227A>G of *TAOK1* (chr17: 27802710) identified by trio WES and the reference variant c.306+468G>T (chr17: 27803257) identified by WGS by Sanger sequencing. **(A)** c.227A>G was found in the proband, but not in either parent. **(B)** c.306+468G>T was detected in the proband and mother, but not in the father.

To validate the *de novo* variant c.227A>G and the reference variant c.306+468G>T, Sanger sequencing was performed for the family (see Supplementary Methods). The results showed that the proband carried c.227A>G but that neither parent did (Figure 3A); c.306+468G>T was found in the proband and mother but not in the father (Figure 3B).

### Droplet Digital PCR

**FIGURE 4:**
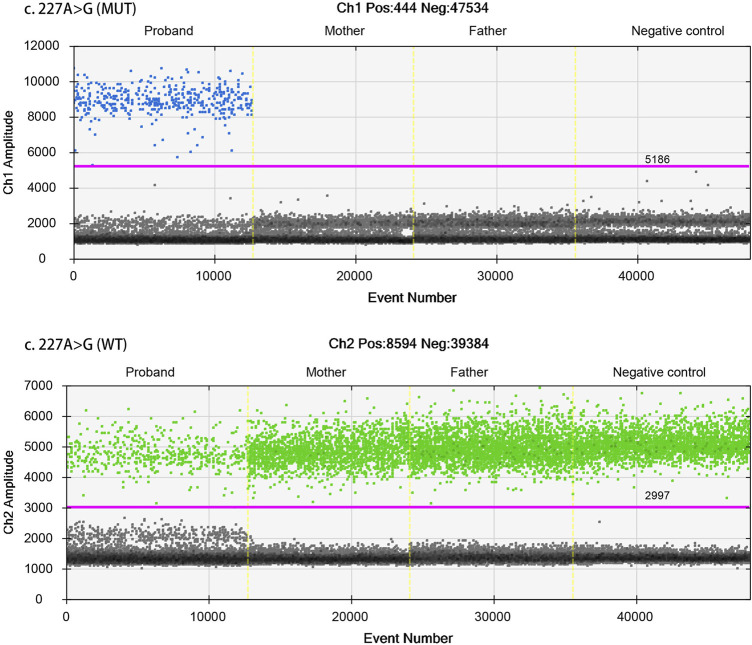
Droplet digital PCR (ddPCR) for mosaic variation detection. The four ddPCRs are divided by vertical dotted yellow lines for the proband, mother, father, and negative control. The pink line is the threshold, above which are positive droplets (blue and green), and below which are negative droplets (gray) without any target DNA. There is no target DNA for the mutant locus c.227A>G in the mother and father (top panel).

ddPCR was used to assess parental mosaicism (see Supplementary Methods). As shown in Figure 4, the peripheral blood samples of the parents did not show mosaicism at the site of the *de novo* variant of *TAOK1*.

**FIGURE 5:**
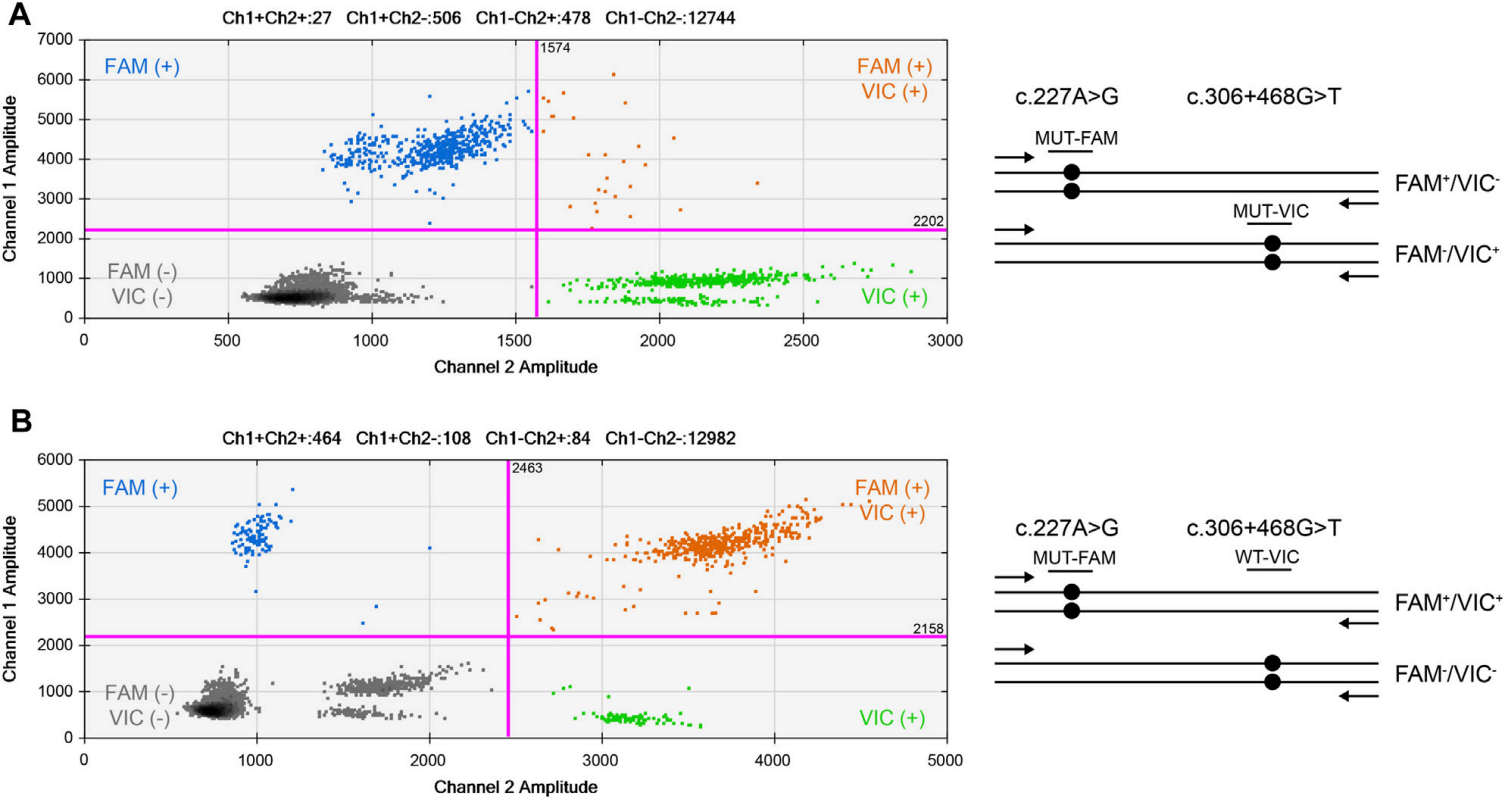
2D cluster plot of droplet fluorescence for the *de novo* locus and reference locus. **(A)** The result of c.306+468G>T mutant and c.227A>G mutant probes. **(B)** The result of c.306+468G>T wild-type and c.227A>G mutant probes. FAM™ positive (Channel 1, mt−) droplets form the top-left blue cluster, HEX™ positive (Channel 2, mt+) droplets form the bottom-right green cluster, negative droplets for both targets form the bottom-left gray cluster, and positive droplets for both targets form the top-right orange cluster.

To determine whether the mutant alleles of the variants, c.227A>G and c.306+468G>T, in the proband were located on the same chromosome, ddPCR was used to verify the phase (see Supplementary Methods). First, the T allele of c.306+468G>T was used as a reference, and phasing was performed. The results showed that these alleles were not located on the same chromosome (Figure 5A). Subsequently, the wild-type allele (G) of c.306+468G>T was used as a reference. The results showed that the G allele of c.306+468G>T and the mutant allele (G) of c.227A>G were located on the same chromosome (Figure 5B). The phasing analysis confirmed that the *de novo* variant c.227A>G derived from the paternal chromosome.

## Discussion

To the best of our knowledge, only six studies have reported variants of *TAOK1* in 40 patients with NDDs (Xie et al., 2016; Dulovic-Mahlow et al., 2019; Satterstrom et al., 2020; Basel-Salmon et al., 2021; van Woerden et al., 2021; Hunter et al., 2022). The phenotypes of the affected individuals are summarized in Table 1. All had NDDs, mainly involving global developmental delay, intellectual disability, hypotonia, and behavior problems, as well as brain MRI abnormalities and eye/visual problems. In this study, the fetus with a variant of *TAOK1* had a dilated left lateral ventricle, and brain MRI imaging in six previously reported postnatal cases revealed dilated lateral ventricles. However, the published studies did not differentiate between unilateral and bilateral ventricular dilation. Therefore, it is uncertain whether variants of *TAOK1* are associated with asymmetric ventricles.

**TABLE 1:** Overview of the identified variants of *TAOK1*.

Patients (Gender)	Chromosome position (GRCh37/hg19)	cDNA change (Amino acid change)	Inheritance	Intellectual disability	Hypotonia	Behavior problems	Brain MRI abnormalities	Eye/visual problems	Pathogenic (ACMG scoring)	Reference
P1 (F)	chr17: 27064286-28761847 × 1	1.69 Mb	*De novo*	+	NR	NR	NR	NR	P (PVS1, PS2, PM2)	Xie et al. (2016)
P2 (M)	chr17: 27861216	c.2442delG (p.Tyr815Ilefs*31)	*De novo*	+	−	+	−	+	P (PVS1, PS2, PM2)	van Woerden et al. (2021)
P3 (M)	chr17: 27818884	c.831+1dupG (p.?)	*De novo*	+	+	+	+	NR	P (PVS1, PS2, PM2)	van Woerden et al. (2021)
P4 (M)	chr17: 27837949	c.1643T>C (p.Leu548Pro)	*De novo*	+	+	+	+	−	LP (PS2, PM2, PP3)	van Woerden et al. (2021)
P5 (F)	chr17: 27822746	c.999+1dupG (p.?)	*De novo*	+	−	−	+	−	P (PVS1, PS2, PM2)	van Woerden et al. (2021)
P6 (M)	chr17: 27844585	c.1819C>T (p.Gln607Ter)	*De novo*	−	+	+	+	−	P (PVS1, PS2, PM2)	van Woerden et al. (2021)
P7 (F)	chr17: 27818877-27818878	c.825_826insCT (p.Lys277Ter)	*De novo*	−	+	−	NR	NR	P (PVS1, PS2, PM2)	van Woerden et al. (2021)
P8 (M)	chr17: 27816684	c.658G>T (p.Glu220Ter)	Maternal	+	−	+	NR	+	P (PVS1, PM2, PP1)	van Woerden et al. (2021)
P9 (M)	chr17: 27849514	c.2125C > T (p.Arg709Ter)	Paternal	−	−	+	−	+	P (PVS1, PM2, PP1)	van Woerden et al. (2021)
P10 (M)	chr17: 27805365	c.449G>T (p.Arg150Ile)	*De novo*	+	NR	NR	NR	NR	LP (PS2, PM1, PM2, PP3)	van Woerden et al. (2021)
P11 (M)	chr17: 27807436	c.500T>G (p.Leu167Arg)	*De novo*	+	−	+	+	−	LP (PS2, PM1, PM2, PP3)	van Woerden et al. (2021)
P12 (M)	chr17: 27849472	c.2083C>T (p.Arg695Ter)	*De novo*	−	+	−	NR	+	P (PVS1, PS2, PM2)	van Woerden et al. (2021)
P13 (F)	chr17: 27805366	c.449+1G>C (p.?)	*De novo*	−	−	−	NR	−	P (PVS1, PS2, PM2)	van Woerden et al. (2021)
P14 (F)	chr17: 27805309	c.393dupT (p.Thr132Tyrfs*19)	*De novo*	+	-	+	−	−	P (PVS1, PS2, PM2)	van Woerden et al. (2021)
P15 (M)	chr17: 27849493	c.2104C>T (p.Arg702Ter)	Unknown	+	+	+	−	+	LP (PVS1, PM2)	van Woerden et al. (2021)
P16 (M)	chr17: 27822689	c.943C>T (p.Leu315Phe)	*De novo*	−	−	+	NR	+	LP (PS2, PM2, PP3)	van Woerden et al. (2021)
P17 (M)	chr17: 27829690	c.1287delA (p.Lys429Asnfs*42)	*De novo*	+	+	+	−	−	P (PVS1, PS2, PM2)	van Woerden et al. (2021)
P18 (F)	chr17: 27802715-27802716	c.232_233delAA (p.Lys78Valfs*20)	*De novo*	+	+	−	−	−	P (PVS1, PS2, PM2)	van Woerden et al. (2021)
P19 (M)	chr17: 27848992-27849799	c.1909-306_2148+262del (p.? [exon 17 deletion])	*De novo*	+	+	−	−	−	P (PVS1, PS2, PM2)	van Woerden et al. (2021)
P20 (F)	chr17: 27816717	c.691A>G (p.Met231Val)	Unknown	+	−	+	−	−	VUS (PM1, PM2, PP3)	van Woerden et al. (2021)
P21 (F)	chr17: 27844579	c.1813C>T (p.Arg605Ter)	Unknown	+	+	−	−	+	LP (PVS1, PM2)	van Woerden et al. (2021)
P22 (F)	chr17: 27080000-29080000 × 1	2 Mb	Unknown	+	+	−	+	-	LP (PVS1, PM2)	van Woerden et al. (2021)
P23 (M)	chr17: 27670438-27934287 × 1	264 kb	Maternal	NR	+	+	−	+	P (PVS1, PM2, PP1)	van Woerden et al. (2021)
P24 (F)	chr17: 27778616	c.50A>G (p.Glu17Gly)	*De novo*	−	+	+	NR	+	LP (PS2, PM2)	Dulovic-Mahlow et al. (2019)
P25 (M)	chr17: 27822638	c.892A>G (p.Lys298Glu)	*De novo*	−	+	+	NR	−	LP (PS2, PM2, PP3)	Dulovic-Mahlow et al. (2019)
P26 (M)	chr17: 27857617	c.2341G>T (p.Glu781*)	*De novo*	+	−	−	NR	−	P (PVS1, PS2, PM2)	Dulovic-Mahlow et al. (2019)
P27 (F)	chr17: 27822660	c.914A>C (p.Asp305Ala)	*De novo*	+	−	−	NR	−	LP (PS2, PM2, PP3)	Dulovic-Mahlow et al. (2019)
P28 (M)	chr17: 27837936	c.1630C>T (p.Gln544*)	*De novo*	−	+	+	NR	−	P (PVS1, PS2, PM2)	Dulovic-Mahlow et al. (2019)
P29 (F)	chr17: 27804704	c.332C>T (p.Ser111Phe)	*De novo*	+	+	−	NR	−	LP (PS2, PM1, PM2, PP3)	Dulovic-Mahlow et al. (2019)
P30 (M)	chr17: 27861140	c.2366_2367insC (p.Leu790Phefs*3)	*De novo*	+	+	+	NR	−	P (PVS1, PS2, PM2)	Dulovic-Mahlow et al. (2019)
P31 (M)	chr17: 27861262	c.2488G>T (p.Glu830*)	*De novo*	−	+	−	NR	−	P (PVS1, PS2, PM2)	Dulovic-Mahlow et al. (2019)
P32 (NR)	chr17: 27778636	c.70C>A (p.Pro24Thr)	*De novo*	+	NR	NR	NR	NR	LP (PS2, PM2, PP3)	Satterstrom et al. (2020)
P33 (NR)	chr17: 27807436	c.500T>G (p.Leu167Arg)	*De novo*	+	NR	NR	NR	NR	LP (PS2, PM1, PM2, PP3)	Satterstrom et al. (2020)
P34 (NR)	chr17: 27822611	c.865G>A (p.Val289Met)	*De novo*	+	NR	NR	NR	NR	LP (PS2, PM2, PP3)	Satterstrom et al. (2020)
P35 (NR)	chr17: 27816682	c.656C>T (p.Ala219Val)	*De novo*	+	NR	NR	NR	NR	LP (PS2, PM1, PM2, PP3)	Satterstrom et al. (2020)
P36 (F)	chr17: 27857424	c.2149-1G>A (p.?)	*De novo*	NR	NR	NR	NR	NR	P (PVS1, PS2, PM2)	Basel-Salmon et al. (2021)
P37 (M)	chr17: 27857479	c.2203delA (p.Arg735Aspfs*6)	Maternal	NR	+	+	+	NR	P (PVS1, PM2, PP1)	Hunter et al. (2022)
P38* (F)	chr17: 27857479	c.2203delA (p.Arg735Aspfs*6)	Maternal	NR	+	+	+	NR	P (PVS1, PM2, PP1)	Hunter et al. (2022)
P39 (M)	chr17: 27778701-27778704	c.132+3_132+6 delAAGT (p.?)	*De novo*	NR	+	+	+	NR	LP (PS2, PM2, PP3)	Hunter et al. (2022)
P40 (F)	chr17: 2 7829727	c.1324C>T (p.Arg442Trp)	*De novo*	NR	+	+	−	NR	LP (PS2, PM2, PP3)	Hunter et al. (2022)
P41 (NA)	chr17: 27802710	c.227A>G (p.Glu76Gly)	*De novo*	NA	NA	NA	+	NA	LP (PS2, PM1, PM2, PP3)	Current study

F, female; M, male; NR, not report; NA, not available; p.?, the effect on protein is unknown; +, present; −, absence; P, pathogenic; LP, likely pathogenic; VUS, variant of uncertain significance; *, Patient 38 is the older sibling of patient 37.

This is the first report of a variant of *TAOK1* in the prenatal stage. We sought to determine whether prenatal *de novo* variants of *TAOK1* can predict the risk of NDDs. To evaluate associations between the *de novo* variant of *TAOK1* we found and phenotypes in the prenatal stage, we compared the pregnancy statuses of the patients with variants of *TAOK1* (Table 2). As shown, few abnormal pregnancy statuses were found. MRI abnormalities in the fetus are relatively prevalent in patients with *TAOK1*-associated NDDs. We suggest that fetuses with brain MRI abnormalities accompanied by *de novo* variants of *TAOK1* have a higher risk for NDDs, and should be carefully managed. Our study not only fills the gap between the variant of *TAOK1* and the prenatal phenotypes but also provides valuable information for disease management, prognosis judgment and prenatal consultation.

**TABLE 2:** Clinical features of patients with *TAOK1* variants.

	Previous Studies	Current Prenatal Case
Gender		
Male	21/36 (58.3%)	NR
Pregnancy status		
Normal	1/22 (4.5%)	Yes
Uncomplicated	13/22 (59.1%)	No
Complicated	1/22 (4.5%)	No
*In vitro* fertilisation	2/22 (9.1%)	No
Polyhydramnios	5/26 (19.2%)	No
Ventricular dilatation	1/22 (4.5%)	Yes
Preeclampsia	1/22 (4.5%)	No
Oligohydramnios	1/22 (4.5%)	No
Pregnancy-induced hypertension	1/22 (4.5%)	Unknown
No prenatal care	1/22 (4.5%)	No
Neurodevelopmental disorder		
Global developmental delay	29/34 (85.3%)	NA
Intellectual disability	24/34 (70.6%)	NA
Hypotonia	22/33 (66.7%)	NA
Behavior problems	21/33 (63.6%)	NA
Brain MRI abnormalities	9/20 (45.0%)	Yes
Eye/visual problems	9/27 (33.3%)	NA

NR, fetal gender in the current case is not reported; NA, the feature is too early to observe in the prenatal case.

As shown in Table 1, *de novo* variants of *TAOK1* have been found in thirty-one of 40 previously reported patients (77.5%). An additional *de novo* variant of *TAOK1* was detected in the fetus in this study. Variants in five (12.5%) affected individuals, P8, P9, P23, P37, and P38, were considered to be inherited from an affected mother or father (Table 1), which are classified as pathogenic herein. The very mild cognitive phenotypes of some affected parents might be explained by incomplete penetrance and variable expressivity (Hunter et al., 2022). Furthermore, no recurrent variants were reported in the region of 17q11.2 (chr17: 27064286-28761847), indicating that *TAOK1* is not prone to hotspot variant, which was also mentioned by van Woerden et al. (2021). The variant identified in this study is located at chr17: 27802710, within the range of previous findings. Based on the protein structure modeling result (Figure 2), p.Glu76Gly is predicted to be located in an alpha-helix structure, and glycine is generally considered to destabilize an alpha-helix. Accordingly, we predicted that this novel missense variant affects protein kinase function, though more functional experiments are needed to validate this assumption.

In this study, the *de novo* variant was confirmed to originate from the paternal chromosome by a ddPCR phasing strategy, consistent with the findings that *de novo* variants arise more frequently in paternal germ cells than in maternal germ cells (Kong et al., 2012; Goldmann et al., 2016). The primarily *de novo* variants on the paternal chromosome could be explained by fundamental differences in germ cell biology in the female and male lineages. Spermatogenesis requires regular mitotic cell divisions of spermatogonial stem cells throughout male reproductive life (Goriely and Wilkie, 2012). However, the influence of maternal chromosomes on *de novo* variants in offspring cannot be ignored (Gao et al., 2019; Goldmann et al., 2019). In recent years, *de novo* variants have been found to be a prominent cause of NDDs, including intellectual disability (ID), autism, and schizophrenia (SCZ) (Veltman and Brunner, 2012; Acuna-Hidalgo et al., 2016). The relationship between paternal-age-related *de novo* variants and the risk for psychiatric and developmental disorders has been assessed, including for autism spectrum disorder (ASD), congenital heart disease (CHD), NDDs with epilepsy (EPI), ID, and SCZ (Taylor et al., 2019). Recurrent risk of a *de novo* variant should be considered if a germline mosaic variant is detected in parental samples, and the sibling recurrent risk can be as low as 0.5% if absent from samples of both parents by highly sensitive screening technology (Wright et al., 2019).

Variants of a gene of uncertain significance should always be classified as having uncertain significance of pathogenicity (Richards et al., 2015). When we obtained the trio WES results in November 2020, the *TAOK1* gene has not been associated with any Mendelian disorder in the OMIM (Online Mendelian Inheritance in Man) database. We further explored research articles and found that Dulovic-Mahlow et al. (2019) first reported eight patients, all with *de novo* variants considered pathogenic due to loss of function of the TAO kinase family. We then evaluated the gene-disease association following the ClinGen Gene-Disease Validity Standard Operating Procedures (Strande et al., 2017), and curated the *TAOK1* gene to “moderate” grade. Finally, the *de novo* variant c.227A>G of *TAOK1* in our case was classified as likely pathogenic (PS2+PM1+PM2+PP3) based on ACMG guidelines (Zhang et al., 2020). A clear understanding of the clinical validity of the gene-disease relationship is critical for accurate interpretation of variants and successful medical decision-making based on genetic testing results. Because of limitations of the prenatal phenotype, accurate genetic variant classification in prenatal diagnosis is especially important. It would benefit from cross-laboratory data sharing and evaluating the strength of a gene-disease relationship based on the ClinGen Gene-Disease Validity Standard Operating Procedures. During the revision of the manuscript, the definitive classification of the gene-disease relationship between *TAOK1* and syndromic intellectual disability was curated by the ClinGen Intellectual Disability and Autism Gene Curation Expert Panel on 4 August 2021. In addition, the *TAOK1* gene was associated with OMIM disease (developmental delay with or without intellectual impairment or behavioral abnormalities, MIM #619575) starting from 19 October 2021. All of these are essential for future work.

## Data Availability

The datasets presented in this study can be found in online repositories. The names of the repository/repositories and accession number(s) can be found below: Genome Sequence Archive (Genomics, Proteomics, and Bioinformatics 2021) in National Genomics Data Center (Nucleic Acids Res 2021), China National Center for Bioinformation/Beijing Institute of Genomics, Chinese Academy of Sciences (GSA-Human: HRA001877) that are publicly accessible at https://ngdc.cncb.ac.cn/gsa-human.
