# Supplementary material for: Paternal De Novo Variant of TAOK1 in a Fetus With Structural Brain Abnormalities
Source: Front Genet. 2022 Jul 19;13:836853. doi: 10.3389/fgene.2022.836853 (PMC9343781; doi:10.3389/fgene.2022.836853)
Supplement: Supplementary file 1 [file DataSheet1.docx]

**Supplemental materials**

**Supplemental methods**

**DNA extraction**

Genomic DNA samples were extracted from whole blood using RelaxGene Blood DNA System (Tiangen, Beijing, China). The quality of genomic DNA was evaluated by NanoDrop 2000 spectrophotometer (Thermo Scientific, Waltham, MA) and agarose gel analysis.

**Chromosomal microarray analysis (CMA)**

Microarray analysis was performed using an Affymetrix Cytoscan 750K GeneChip. Data analysis was performed using the Chromosome Analysis Suite (ChAS) 4.1 software.

**Trio whole exome sequencing (Trio-WES)**

Genomic DNA was sheared into proper pieces (150-200bp) by a Covaries ultrasonicator. The target genomic regions were captured by hybridizing the genomic DNA sample library with the xGen® Exome Research Panel v1.0 (IDT, USA). High-throughput sequencing was then performed on Illumina NovaSeq6000 (Illumina, San Diego, CA, USA) with 150 bp paired-end reads.

The clean reads were then aligned against human reference (GRCh37) with BWA^[1]^. SNPs/Indels were discovered by the HaplotypeCaller tool of GATK after necessary post processes on primary alignment including duplicated reads removing, realignment and base recalibrating^[2]^. VEP was employed to identify the effect of all discovered variants followed by variant annotation with AnnoVar^[3]^. Notably, each variant was compared against public databases, including gnomAD, 1000 genomes project, NHLBI Exome Sequencing Project 6500 (ESP6500), and Exome Aggregation Consortium (ExAC), to achieve allele frequency in the general population. To identify the known and reported pathogenic variants, each variant was also compared against ClinVar (https://www.ncbi.nlm.nih.gov/clinvar) and HGMD(www.hgmd.cf.ac.uk). In terms of possible influence on the protein function, variants were evaluated by popular prediction tools, including SIFT, DANN and REVEL^[4-6]^. Based on the variant annotations, a series of filtering strategies were applied to identify candidate SNVs/Indels associated with EEs, which were described previously^[7]^.

CNVs were detected by CNVkit^[8]^. To create a stable and reliable CNV reference, in-house samples over the same sequencing protocol were selected for reference training in an iterative manner. Specifically, a set of 80 samples was used to create an initial CNV reference. CNV calling process was then run over this reference for each sample in the training set. Samples with any CNV events larger than 1Mbp were excluded from the next iteration. In the next iteration, new samples were added in to make up the training set to be 80 samples. The iteration process was ended once no less than 50 samples were qualified. During the reference creation process, circular binary segmentation (CBS) algorithm was chosen for CNV event segmentation, and the threshold parameter for copy number calling was set to be “-1.6,-0.8,0.5,1”. For better visualization investigation of CNV events, a tool was designed to plot the copy number aligned with B-allele frequencies along with chromosome coordinates. The copy number was shown as log2 ratio obtained from bin level and segmented level CNV calling results. The B-allele frequencies were computed by the samtools mpileup tool. AnnotSV and its annotation databases was locally installed to annotate detected CNV events for each tested samples for following clinical interpretation^[9]^.

**Whole genome sequencing (WGS)**

Genomic DNA was fragmented by a Covaris ultrasonicator, followed by library preparation. High-throughput sequencing was then performed on Illumina NovaSeq6000 (Illumina, San Diego, CA, USA) platform with 150 bp paired-end reads. Bioinformatic analysis was carried out with public software and a self-developed pipeline. Specifically, all cleaned data after trimming were aligned against the human reference genome build hg19 using BWA^[1]^. Short variants (SNPs and indels) were then discovered by HaplotypeCaller of GATK, followed by variant annotation through ANNOVAR^[10]^, with the integration of customized databases such as ClinPred. Analysis was performed on all sequenced genomic regions, including both exonic and intronic regions, especially the upstream and downstream of the mutation identified by trio WES.

**Sanger sequencing**

Sanger sequencing was arranged to confirm the mutations identified by trio WES and WGS. Primers (Supplemental Table S1) were designed by Primer 5. PCR was conducted with TaKaRa Taq DNA Polymerase and premix under the following conditions: initial denaturation at 95 °C for 5 min, followed by 32 cycles at 95 °C for 30 s, 58 °C for 30 s and 72 °C for 40 s and a final hold at 72 °C for 10 min. PCR products were purified and sequenced using an ABI 3730 DNA Analyzer with the BigDye™ Terminator Cycle Sequencing Kit (Applied Biosystems, Foster, CA, USA).

Supplemental Table S1 **Primers for Sanger sequencing**

| **Name** | **Primers** |
| --- | --- |
| TAOK1-c.227A>G -PA0004679F | GTAAAACCAAGCAAAGTTGCATGT |
| TAOK1-c.227A>G -PA0004679R | ACATATGCTAACTTTCAACCTCAAGT |
| TAOK1-REF -PA0004978F | GCATGGGTTGGTATTTGTTC |
| TAOK1-REF -PA0004978R | CCTCCTATGTGCCAGGTATT |

**Droplet Digital PCR (ddPCR)**

*Detection of Mosaic Mutation*

Droplet digital reactions were carried out in a 20µl volume consisted of 2ul genomic DNA (fetal amniotic fluid, father, mother, black control, separately), 2ul primers (TAOK1-c.227A>G-F and TAOK1-c.227A>G-R), 2ul fluorescent hydrolysis probes (TAOK1-c.227A>G-w-P and TAOK1-c.227A>G-m-P) (Supplemental Table S2) and 10 ul ddPCR SuperMix for Probes (No dUTP) (Bio-Rad). Droplets were generated using the Qx200 Droplet Generator, and cycled in a C1000 thermal cycler (Bio-Rad) following: 1) 95°C for 10 min (1 cycle), (94°C for 30 s, 60°C for 1 min) for 40 cycles, 98°C for 10 min (1 cycle), 4°C for 1 min (1 cycle). The droplets were read using a QX200 droplet reader and data was analyzed using QuantaSoft v1.7 (Bio-Rad).

Supplemental Table S**2 Primers and probes for Droplet Digital PCR**

| **Name** | **Primer/Probe Sequence** | **5'** | **3'** |
| --- | --- | --- | --- |
| TAOK1-c.227A>G-F | TCTCCCCACAGAAATGGCAG |  |  |
| TAOK1-c.227A>G-R | TACTGTTGGGATGTTTTATTCTTTGTAGA |  |  |
| TAOK1-c.227A>G-w-P | ATTATTAAGGAAGTCAAGTTT | VIC | MGB |
| TAOK1-c.227A>G-m-P | TTATTAAGGGAGTCAAGTTT | FAM | MGB |
| TAOK1-REFG>T-F | CCTTGGTCGAATAACACTAAAGATAGAG |  |  |
| TAOK1-REFG>T-R | ATTCCTGGTAACAAAAGGGCAA |  |  |
| TAOK1-REFG>T-w-P2 | AGAAGTAGAAATTTATTTTGCCC | FAM | MGB |
| TAOK1-REFG>T-m-P2 | AAGAAGTATAAATTTATTTTGCCC | VIC | MGB |
| TAOK1-REFG>T-w-P2-1 | AGAAGTAGAAATTTATTTTGCCC | VIC | MGB |

*Droplet Digital PCR Phasing*

Droplet digital reactions were carried out in a 20µl volume consisted of 2ul genomic DNA (fetal amniotic fluid), 4ul primers (TAOK1-c.227A>G-F, TAOK1-c.227A>G-R, TAOK1-REFG>T-F, TAOK1-REFG>T-R), 2ul fluorescent hydrolysis probes (TAOK1-c.227A>G-m-P, TAOK1-REFG>T-m-P2) (Supplemental Table S2) and 10 ul ddPCR SuperMix for Probes (No dUTP) (Bio-Rad). Droplets were generated using the Qx200 Droplet Generator, and cycled in a C1000 thermal cycler (Bio-Rad) following: 1) 95°C for 10 min (1 cycle), (94°C for 30 s, 60°C for 1 min) for 40 cycles, 98°C for 10 min (1 cycle), 4°C for 1 min (1 cycle). The droplets were read using a QX200 droplet reader and data analyzed was using QuantaSoft v1.7 (Bio-Rad).

In order to verify the accuracy of the result, we repeated the above experiments with probes (TAOK1-c.227A>G-m-P, TAOK1-REFG>T-w-P2-1) (Supplemental Table S2).

**References**

[1] Li H, Durbin R. Fast and accurate short read alignment with Burrows-Wheeler transform. Bioinformatics. 2009. 25(14): 1754-60.

[2] McKenna A, Hanna M, Banks E, et al. The Genome Analysis Toolkit: a MapReduce framework for analyzing next-generation DNA sequencing data. Genome Res. 2010. 20(9): 1297-303.

[3] McLaren W, Gil L, Hunt SE, et al. The Ensembl Variant Effect Predictor. Genome Biol. 2016. 17(1): 122.

[4] Ng PC, Henikoff S. SIFT: Predicting amino acid changes that affect protein function. Nucleic Acids Res. 2003. 31(13): 3812-4.

[5] Quang D, Chen Y, Xie X. DANN: a deep learning approach for annotating the pathogenicity of genetic variants. Bioinformatics. 2015. 31(5): 761-3.

[6] Ioannidis NM, Rothstein JH, Pejaver V, et al. REVEL: An Ensemble Method for Predicting the Pathogenicity of Rare Missense Variants. Am J Hum Genet. 2016. 99(4): 877-885.

[7] Zhou P, He N, Zhang JW, et al. Novel mutations and phenotypes of epilepsy-associated genes in epileptic encephalopathies. Genes Brain Behav. 2018. 17(8): e12456.

[8] Talevich E, Shain AH, Botton T, Bastian BC. CNVkit: Genome-Wide Copy Number Detection and Visualization from Targeted DNA Sequencing. PLoS Comput Biol. 2016. 12(4): e1004873.

[9] Geoffroy V, Herenger Y, Kress A, et al. AnnotSV: an integrated tool for structural variations annotation. Bioinformatics. 2018. 34(20): 3572-3574.

[10] Wang K, Li M, Hakonarson H. ANNOVAR: functional annotation of genetic variants from high-throughput sequencing data. Nucleic Acids Res. 2010. 38(16): e164.
